# Successful Fitting of a Complete Maxillary Denture in a Patient with Severe Alzheimer's Disease Complicated by Oral Dyskinesia

**DOI:** 10.1155/2016/4026480

**Published:** 2016-10-16

**Authors:** Hiromitsu Morita, Akie Hashimoto, Ryosuke Inoue, Shohei Yoshimoto, Masahiro Yoneda, Takao Hirofuji

**Affiliations:** ^1^Section of General Dentistry, Department of General Dentistry, Fukuoka Dental College, Fukuoka 814-0193, Japan; ^2^Special Patient Oral Care Unit, Kyushu University Hospital, Fukuoka 812-8582, Japan

## Abstract

There is an increasing population of elderly patients suffering from Alzheimer's disease (AD), the most common form of dementia. In dentistry, a critical problem associated with these patients is the use of a new denture, as AD patients often refuse dental management and are disturbed by minor changes in their oral environment. Some AD patients have further complications associated with oral dyskinesia, a movement disorder that can make dental management difficult, including the stability of a complete denture. In this case, we successfully fitted a complete maxillary denture using modified bilateral balanced occlusion after multiple tooth extractions under intravenous sedation in a 66-year-old woman with severe AD complicated by oral dyskinesia. Following treatment, her appetite and food intake greatly improved. Providing a well-fitting complete denture applied by modified bilateral balanced occlusion, which removes lateral interference using zero-degree artificial teeth for movement disorder of the jaw in patients with severe AD complicated by oral dyskinesia, helps improve oral function.

## 1. Introduction

Alzheimer's disease (AD) is the most common form of dementia, and patients with severe AD experience problems associated with cognitive function such as aphasia, apraxia, agnosia, and disorientation, making daily social activities difficult [1–3]. Worldwide, 47.5 million people suffered from dementia in 2015, and there are 7.7 million new cases every year [4]. The communication ability of patients with severe AD is poor, and dental management for them is critically restricted [1–3]. It is therefore difficult for such patients to receive both dental treatment and periodic dental follow-up, including oral care. A further difficulty is that some patients are disturbed by minor changes in their oral environment, such as a new denture, and struggle to adapt to the change [1]. Patients with severe AD often experience oral dyskinesia, which makes it more difficult not only to manage dental treatment but also to stabilize dentures [1–3].

Here we report a patient with severe AD, possibly showing signs of agnosia rather than impairment in adaptation, whose eating disorder, caused by severe periodontitis and oral dyskinesia, greatly improved following multiple tooth extractions under intravenous sedation (IVS) and the making and placement of a new, functional, stabilized, and well-fitting complete maxillary denture with bilateral balanced occlusion using zero-degree artificial teeth.

## 2. Case Presentation

A 66-year-old woman with severe AD complicated by oral dyskinesia was referred for dental treatment by her neurologist. She was unable to eat any solid food because of limited mobility of her maxillary teeth as a result of severe periodontitis. Because of her oral condition and subsequent lack of food, her body weight had decreased by ~5 kg within 2 months. Her husband was concerned for her health and hoped that her oral function would improve with the extraction of her diseased teeth and replacement with a removable denture. Written informed consent was obtained from the patient's husband for publication of this case report following the Ethical Guidelines of Kyushu University Hospital.

Her medical history revealed that she developed severe AD in 2004. Her medical condition suddenly worsened in 2006, resulting in severe cognitive impairment. Since 2011, she has been medicated with NMDA inhibitors and cholinesterase inhibitors. She has continued to receive regular medical treatment.

Her dental history revealed that she routinely visited a local dental office for oral care and maintenance until July 2012. Her family dentist recommended extraction of all her remaining maxillary teeth and asked her husband to consent to extractions under IVS in hospital because of the difficulty of conducting the dental procedure while she was awake. The patient's neurologist wrote a referral for her, and her husband brought her to our department, the Special Patient Oral Care Unit, Kyushu University Hospital, in October 2012. When she presented at our department, she exhibited typical features of AD, such as aphasia, gait apraxia, and disorientation. Her height and weight were 151 cm and 46 kg, respectively. Her serum albumin was 3.8 g/dL. She could not walk by herself and needed her husband's assistance. Her cognitive function and activities of daily living using the functional independence measure [5] were 26 points (maximum possible score: 126, minimum possible score: 18). She frequently vocalized meaningless words and could not speak any meaningful words or understand any of our instructions. As she could not sit still, it was impossible to take X-ray images of her teeth. We were able to see and touch inside her mouth only when her husband held her face and opened her mouth gently in semi-Fowler's position. If we tried to insert any instrument into her mouth by ourselves, she brushed our hands away. With her husband's help, we were able to briefly assess her oral condition and established the necessity for multiple extractions of her remaining maxillary teeth and fabrication of a complete denture. We initially planned to take impression before extraction to maintain her original maxilla-mandibular horizontal relationship under IVS. However, this was not possible as the horizontal position had collapsed from severe lateral mobility of the metal bridge between 13 and 17. To avoid accidents and the need for restraint and to maintain the patient's dignity during dental treatment, we decided to use IVS for oral examination and dental management, with the approval of her husband. In addition, her medical doctor checked her general health and approved the use of IVS.

**Figure 1:**
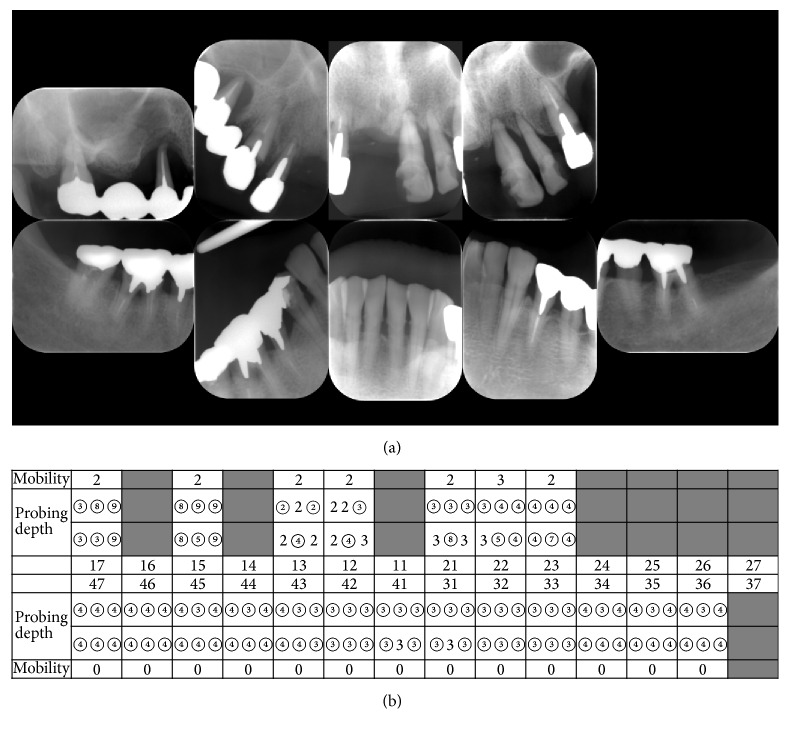
(a) X-ray images and (b) periodontal chart at initial dental visit. Circle on number indicates bleeding on probing.

On the first day of her dental treatment, we planned to undertake a complete examination, tooth scaling, multiple tooth extractions, and an impression for her denture under IVS. The IVS procedure involved initial administration of 4 mg midazolam, and once the patient was drowsy, we added propofol for maintenance. Under IVS, we first took X-rays and checked the probing depth and mobility of the remaining teeth. We observed severe periodontitis of all maxillary teeth (12, 13, 15, 17, 21, 22, and 23) and moderate periodontitis of all mandibular teeth (Figure 1). We scaled the teeth and subsequently extracted the maxillary teeth. All sockets were sutured using bioabsorbable thread. Using alginate in a custom impression tray, we took impressions of the maxillary arch and the opposing mandibular teeth for a complete maxillary denture. We finished the dental treatment after confirming that there was no bleeding from the sockets. The duration of the dental treatment was 57 min, and the duration of the anesthesia was 1 h and 20 min. In total, 49 mg of propofol was used. There were no perioperative complications.

To help stop bleeding and as an imitation trial of the complete denture, we made a temporary base plate that covered her maxilla with tray resin and a plaster model, which duplicated for her teeth impression for the denture until recovery from IVS. After recovery from IVS, we tried fitting the base plate and relined with tissue conditioner. Surprisingly, she easily accepted it. Following this success, we sought to fabricate a new complete denture, as requested by her husband.

One day after extraction, there was no bleeding inside the patient's mouth, and she was still wearing the base plate without any signs of refusal. We removed the temporary base plate, checked to ensure that there was indeed no bleeding, washed her sockets with saline, and then refitted the base plate to her maxilla. We provided her husband with instructions for care, which included removing and washing the base plate after every meal and before the patient went to sleep. This was done as training before use of the complete denture.

**Figure 2:**
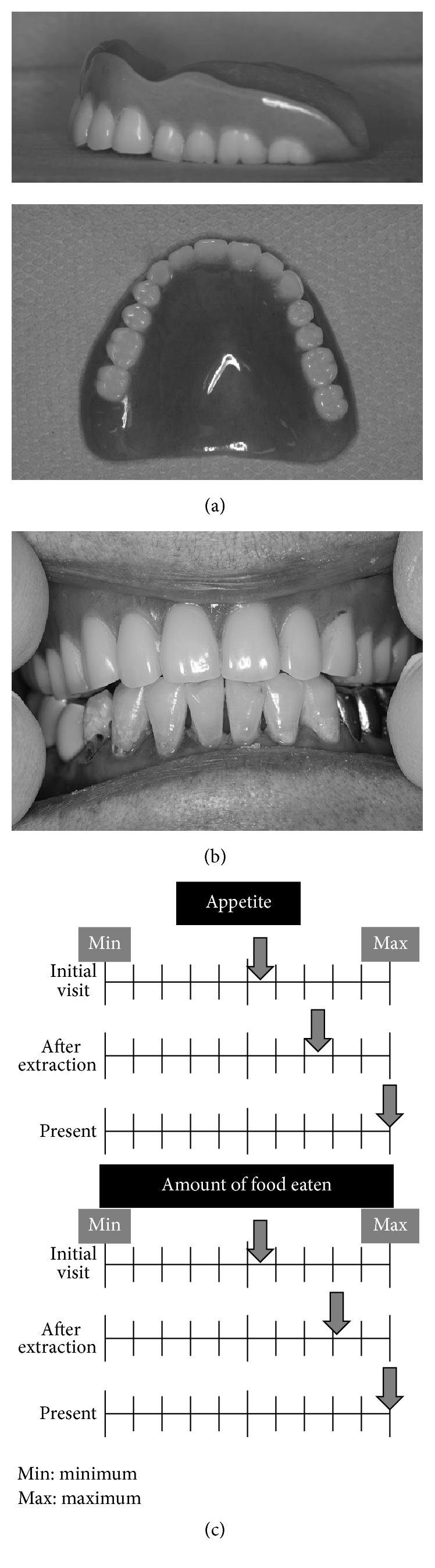
(a) Lateral and occlusal views of the complete maxillary denture. (b) Frontal view of the complete maxillary denture. (c) Visual analogue scale of appetite and amount of food eaten at the time of the initial visit, after extraction, and posttreatment, drawn by the patient's husband.

One week after the extractions, we attempted to take a bite using Willis's method in semi-Fowler's position, opening the patient's mouth with her husband's help. Maxilla-mandibular horizontal position was achieved by moving the patient's chin forward using our hands. After a trial fitting, we fabricated her initial denture. To avoid instability caused by lateral cuspal interference because of her oral dyskinesia, we used zero-degree artificial teeth (Figure 2(a)). On inserting the new complete maxillary denture, we found that the mucosal surface of the denture was ill-fitting, because the impression had been taken immediately after multiple tooth extractions. To improve the fit, we relined the denture using low-flow tissue conditioner, COE-COMFORT™ (GC, Tokyo, Japan) and held her jaw gently for a while in semi-Fowler's position because of her oral dyskinesia. When we tried fitting the new denture, she initially hesitated to wear it; however, she accepted it soon after it was relined, similar as to when she wore the base plate to stop bleeding. After occlusal adjustment of the denture using articulating paper to remove lateral cuspal interference when she was awake, we instructed her husband on how to manage the denture, including insertion, removal, and cleaning, and the patient went home wearing the denture.

One week later, she visited our department for a denture adjustment. Her husband told us that she had not complained or indicated that the denture caused any pain or discomfort and had not tried to remove the denture once inserted. Her husband reported that she was able to eat soft solid food such as grilled fish, and her appetite had increased. She visited our unit 2 days per week for a denture adjustment and relining over several weeks. Three months after the extractions, the sockets were almost completely healed and bone had recovered. We performed dynamic impressions for a week using the same tissue conditioner and temporarily kept her denture for relining, choosing an indirect procedure in the dental laboratory to avoid a poor result because of her oral dyskinesia and to avoid accidental swallowing of the reline materials. After relining and occlusal adjustment of the denture, she was again happy to wear the denture (Figure 2(b)).

The patient has since visited the department for oral hygiene management and denture adjustment, including partial direct rebasing around the sockets several times. Currently, she routinely receives oral examinations and hygiene management. She can eat almost all foods such as vegetables, meat, and fish, and her body weight has increased by 5 kg (now up to 51 kg) and her serum albumin level has also improved (4.3 mg/dL). From discussion with the patient's husband's and examining a visual analogue scale drawn by him, it is apparent that her appetite and food intake have greatly improved (Figure 2(c)).

## 3. Discussion

We successfully performed multiple tooth extractions under IVS and fabricated a stable complete maxillary denture using zero-degree artificial teeth for a patient with severe AD complicated by oral dyskinesia. The treatment appears to have improved both the patient's oral function and her appetite and she has gained weight.

Following multiple tooth extractions, and being considerate of the patient's dignity and at the request of the patient's husband, we initially fitted a base plate. When we observed that she easily accepted the base plate and showed no signs of discomfort or stress, we took this as a sign that a complete denture may be possible. As it was requested by the patient's husband, we began the process of making and fitting a new complete denture.

We initially referred to the report by Fujisawa et al. in which they described successful multiple tooth extractions using IVS and the fitting of a new complete denture in a patient with severe AD [6]. We safely performed dental treatments such as probing, scaling, tooth extraction, and denture impressions under IVS as outlined in previous reports and reviews [1–4, 6–8] to minimize the risk of adverse effects.

An additional problem for our patient was that she suffered from oral dyskinesia, a jaw movement disorder, which is a complication of severe AD. This condition makes it more difficult to stabilize a complete denture. After referring to reports about the fabrication of prostheses for patients with Parkinson's disease complicated by oral dyskinesia, we applied modified bilateral balanced occlusion using zero-degree artificial teeth to avoid interference by the molar cusps during lateral movement [9, 10]. We succeeded in stabilizing the complete maxillary denture in this case. Accordingly, we consider that using zero-degree artificial teeth in complete dentures for patients with oral dyskinesia is useful. As a result of the dental management described above, and with the help of her husband, the patient's appetite and food intake have greatly improved and she has gained weight.

Interestingly, from our clinical observations, it appears that the patient forgot that she was wearing a denture after the fitting of the new complete maxillary denture, possibly suggestive of agnosia. Although it is known that minor changes in the oral environment, such as the fitting of a new denture, can cause distress to AD patients because of their impaired ability to adapt, the patient in this case easily accepted the new denture.

It is our suggestion that fabricating a painless, well-fitting complete denture using modified bilateral occlusion with zero-degree artificial teeth in patients with severe AD with oral dyskinesia may help improve the food intake in such patients.
